# Comparison of Acute Stroke Outcomes Between Code Trauma vs Code Stroke Activations

**DOI:** 10.5811/westjem.48925

**Published:** 2025-12-26

**Authors:** Jacob Brown, Mallory Jebbia, Esther Lee, Albert Kazi, Aaron Strumwasser, Byan Love, John Woods, Babak Khazaeni

**Affiliations:** * Desert Regional Medical Center, Department of Emergency Medicine, Palm Springs, California; † Desert Regional Medical Center, Department of Surgery, Palm Springs, California

## Abstract

**Introduction:**

Patients with acute stroke may occasionally present as trauma activations, particularly after being found down or sustaining falls. This atypical presentation can delay diagnosis and treatment. Our objective in this study was to compare time to brain imaging, use of reperfusion therapies, and clinical outcomes, including discharge disposition and mortality, between patients with acute stroke presenting as code trauma activations and those presenting as code stroke activations.

**Methods:**

We conducted a retrospective review of all trauma activations at our Level I trauma center from January 2018–December 2024. Patients diagnosed with acute stroke on initial trauma imaging after trauma evaluation formed the code trauma activation (CTA) group. These patients were compared to all patients diagnosed with acute stroke after a code stroke activation (CSA) in 2024. The primary outcome was door-to-imaging time; secondary outcomes included door-to-intervention time, discharge disposition, and mortality.

**Results:**

There were 208 CSA patients and 198 CTA patients. The CTA patients were older (75.3 vs 70.3 years of age, *P* < .001) and had a higher percentage of hemorrhagic stroke (43.9% vs 14.4%, *P* < .001). The CTA patients had a higher National Institutes of Health Stroke Scale score (14.44 vs 9.67, *P* < .001). Despite minimal injuries (mean Injury Severity Score 3.3), CTA patients experienced longer times to initial brain imaging (47.4 vs 24.8 minutes, *P* < .001). Mean door-to-thrombolysis (50.3 vs 43.7 minutes, *P* = .19) and door-to-puncture time (98 vs 82 minutes, *P* =.18) did not differ significantly. The CTA patients had lower rates of discharge home (23.2% vs 42.8%, *P* < .001) and higher mortality (24.2% vs 12%, *P* < .001). On multivariate analysis, trauma activation itself was not independently associated with mortality (OR 1.57, CI, 0.53–4.27, *P* =.42). Age, stroke severity scores, hemorrhagic stroke, and early imaging were independently associated with mortality after acute stroke.

**Conclusion:**

Acute stroke patients presenting as trauma activations face significant delays in imaging and lower rates of thrombolytic treatment, despite low injury burden. While trauma activation designation was not independently associated with mortality, delays in imaging and higher hemorrhage prevalence were strongly linked to worse outcomes. These findings highlight modifiable workflow opportunities, particularly streamlined imaging and early stroke recognition in low-impact trauma presentations, to improve delivery of care.

## INTRODUCTION

Stroke remains a major public health burden in the United States with approximately 795,000 cases reported annually.^1^ In 2021, the stroke-related death rate was 41.1 per 100,000 population, reflecting an 8.4% increase from 2011.^1^ Of all strokes, 87% are ischemic, 10% are intracerebral hemorrhages (ICH), and 3% are subarachnoid hemorrhage.^1^ The clinical presentation of stroke varies significantly depending on the affected vascular territory. Middle cerebral artery strokes, which account for roughly 50% of ischemic strokes, typically present with hemiparesis, neglect, and visual field deficits.^2^ Anterior cerebral artery strokes often cause contralateral lower extremity weakness, while posterior cerebral artery or vertebrobasilar system strokes may present with ataxia or vertigo.^3^–^5^ Given the sudden onset and disabling nature of these symptoms, it is not difficult to imagine how a stroke could precipitate a traumatic event, such as a fall while walking, a motor vehicle collision, or an accident while operating machinery. When these patients are found down, especially if there are signs of head trauma, they may be brought to the emergency department (ED) as a trauma activation.

Time is critical in acute stroke care. Large vessel occlusions are estimated to result in the loss of 1.9 million neurons per minute, translating to 3.1 weeks of neurologic aging for every minute of treatment delay.^6^ Current American Stroke Association guidelines emphasize expedited imaging and intervention, targeting brain imaging (door to imaging) within 20 minutes of arrival and thrombolytic administration (door to needle) within 60 minutes. Fibrinolysis remains effective up to 4.5 hours after last known well time, while mechanical thrombectomy may be performed up to 24 hours from last known well time.^7^

In contrast, trauma activations necessitate a more comprehensive initial evaluation based on Advanced Trauma Life Support (ATLS) principles. Primary and secondary surveys, extended focused assessment with sonography for trauma, chest radiograph, pelvis radiograph, and stabilization interventions are typically completed prior to computed tomography (CT).^8^ For patients with relatively minor traumatic injuries but significant neurologic deficits, subtle signs of acute stroke may be underappreciated, leading to critical delays in diagnosis and treatment.

At the health system level, door-to-imaging and door-to-needle time are proven, actionable system performance metrics. Studies have shown that standardized bundles including prehospital notification, direct to CT, and parallel workflows can reliably shorten treatment times across hospitals.^9^,^10^ Earlier reperfusion translates to better functional outcomes, with some studies estimating weeks to months of disability-free life gained for minutes saved in treatment.^11^

Strokes masquerading as trauma pose a unique diagnostic and operational challenge. Although trauma pathways prioritize rapid imaging, the inherent workflow delays compared to stroke activations may impact timely reperfusion therapy. At our institution, parallel activation systems exist for trauma (code trauma) and stroke (code stroke). Our objective in this study was to compare time to brain imaging, use of reperfusion therapies, and clinical outcomes, including discharge disposition and mortality, between patients with acute stroke presenting as code trauma activations and those presenting as code stroke activations. We hypothesized that patients with stroke initially evaluated under the trauma pathway experience delays in brain imaging leading to delayed treatment and higher mortality compared to those triaged through the dedicated stroke pathway.

**Population Health Research Capsule**

What do we already know about this issue?

*Stroke patients mis-triaged as trauma activations may face imaging delays, missed thrombolysis windows, and worse outcomes, but data are limited*.

What was the research question?

*Do stroke patients presenting as a trauma activation differ in time to brain imaging, treatment, and outcomes compared to code stroke patients?*

What was the major finding of the study?

*Code trauma vs code stroke patients: time to brain imaging 47 vs 25 minutes, thrombolytic use 4% vs 26%, mortality 24% vs 12% (P < .001)*.

How does this improve population health?

*Findings highlight workflow changes to speed imaging in atypical stroke presentations, improving access to timely treatment and outcomes*.

## METHODS

After institutional review board approval was obtained, we retrospectively reviewed all adult trauma patients ≥ 18 years of age at our Level I trauma center who were evaluated between January 2018–December 2024. We included all patients who were diagnosed with a cerebrovascular accident on CT during their initial workup. These patients comprised our first group, the code trauma activation (CTA) group. We excluded atients who were evaluated as a trauma consult. We then queried the code stroke hospital database for all patients evaluated as a code stroke and confirmed to have an acute stroke without evidence of traumatic intracranial hemorrhage on CT during 2024. These patients comprised our second group, the code stroke activation (CSA) group. We excluded patients seen as a stroke consult.

To be included in either group patients were diagnosed with acute stroke on initial ED imaging (non-contrast head CT and/or CT angiogram of the head/neck). Hemorrhagic stroke was defined by acute blood on non-contrast CT and determined by radiology to have a non-traumatic pattern. Acute ischemic stroke was defined by radiology-reported acute ischemic changes on non-contrast CT and/or CT angiogram evidence of large-vessel occlusion consistent with the clinical syndrome. We excluded cases with normal initial CT/CT angiogram that were diagnosed on later or alternative imaging. The primary outcome was time to initial brain imaging. Time to brain imaging was defined as time from arrival in the ED to the first time stamp on the non-contrast head CT acquisition, which even in the case of CT angiogram head, a non-contrast run was performed first. Imaging protocols for acute stroke and trauma evaluations remained standardized across the study period. Scanner location and availability did not vary substantially during the study period; a dedicated CT scanner adjacent to the ED was consistently available for both trauma and stroke activations. The secondary outcomes were door-to-intervention time, discharge disposition, and mortality. Trauma activations were conducted in accordance with departmental (ATLS-based) policy, including primary and secondary surveys with typical adjuncts including ultrasounds, chest radiographs, and pelvis radiographs prior to CT when indicated. A formal audit of each chart for strict compliance was not performed at part of the retrospective review.

Demographic data points collected included age and sex. Characteristics including stroke type (hemorrhagic vs ischemic), National Institutes of Health Stroke Scale (NIHSS) score, and mode of hospital arrival were recorded for each group. For the CTA group, injury data collected included mechanism of injury, level of trauma activation (1, 2 or 3), type of injury (fracture type, solid organ injury, or facial soft tissue injury including face or scalp laceration, hematoma, or abrasion), Injury Severity Score (ISS), and total Glasgow Coma Scale (GCS) score on arrival. For each group we recorded and compared time to initial brain imaging, as well as time to interventions such as thrombolytic therapy and thrombectomy. Additional outcomes measured included discharge disposition and length of stay (LOS).

We performed all bivariate analyses with SPSS Statistics for Windows v29 (IBM Corp., Armonk, NY). A Mann-Whitney *U* test was used to compare continuous variables and a chi-square was used to compare categorical variables in the bivariate analysis. We presented categorical data as percentages and continuous data as a mean with standard deviation. We then performed a multivariable logistic regression analysis to determine the risk of mortality for patients with acute stroke arriving as a code stroke activation vs code trauma activation. We adjusted for potential confounders, which were selected based on discussion among coauthors, review of the literature, and identification of univariate, statistically significant differences between proposed confounding variables. These included age, presence of ≥ 2 comorbidities (including hypertension, diabetes mellitus, chronic obstructive pulmonary disease, congestive heart failure, chronic kidney disease, end- stage renal disease, and atrial fibrillation), early imaging (time to CT < 20 minutes), and type of stroke (ischemic vs hemorrhagic). *P*-values were defined as statistically significant if < .05.

This study adheres to the Strengthening the Reporting of Observational Studies in Epidemiology (STROBE) guidelines and followed key methodological principles outlined by Worster et al for medical record review studies in emergency medicine, including the use of clearly defined inclusion and exclusion criteria, and predefined data definitions to minimize bias.^12^

## RESULTS

A total of 208 patients were activated as a code stroke, which was confirmed on CT imaging, while 198 patients were activated as a code trauma but were diagnosed with an acute stroke on imaging. The CTA patients were older (75.3 vs 70.3 years of age, *P* < .001). There was no difference in sex between the two groups (52.5% male vs 59.6% male, *P* = .63). The CTA patients had a higher incidence of hemorrhagic stroke (43.9% vs 14.4%, *P* < .001). The CTA patients were more likely to arrive by ambulance (93.4% vs 79.3%) while CSA patients had a higher percentage arrive by private vehicle (4.0% vs 17.3%, *P* <.001). The CTA patients had a higher average NIHSS score (14.44 vs 9.67, *P* < .001) (Table 1).

We recorded injury patterns for the 198 patients in the CTA group, with the primary mechanism being falls (97%), followed by motor vehicle collision (3%). Of the CTA patients, 40.9% were activated as a level 1 trauma, 41.9% were activated as a level 2, and 17.2% as a level 3 trauma. Recorded injuries were predominantly facial abrasions/hematomas (33.3%), followed by extremity fractures (2.5%), and spinal fractures (2.5%). Two patients had low-grade splenic lacerations (1.0%). The average ISS for CTA patients was 3.3, and the average initial total GCS was 11.7. Of patients arriving as a CTA, 42.4% underwent non-contrast CT head for trauma and stroke protocol imaging during the initial imaging session (Table 2).

The mean time to brain imaging was significantly higher in the CTA group (47.4 vs 24.8 minutes, *P* < .001). More patients in the CSA group underwent thrombolytic therapy (3.6% vs 25.8%, *P* < .001). Of the 107 ischemic stroke patients in the CTA who did not receive thrombolytics, only 24 (22.5%) had a documented contraindication to thrombolytics therapy. Mean door-to-thrombolytics time tended to be higher in the CTA group, but this did not reach statistical significance (50.3 vs 43.7 minutes, *P* = .19). There was no difference in the percentage of patients who underwent thrombectomy in each group (11.1% vs 16.8%, *P* = .13). Mean door-to-puncture time tended to be higher in the CTA group without statistical significance (98 vs 82 minutes, *P* = .18). Disposition from the hospital showed fewer patients in the CTA group going home (23.2%), more going to a skilled nursing facility (39.4%), more going to hospice (6.6%), and fewer leaving against medical advice (2.0%) compared to CSA patients (home, 42.8%; skilled nursing facility, 35.1%; hospice, 1.4%; and against medical advice, 5.3%, *P* < .001). Mortality was higher in the CTA group (24.2% vs 12.0%, *P* < .001) (Table 3). A posthoc power analysis demonstrated our sample size provided > 80% power to detect a > 15- minute difference in mean door-to-imaging time between two groups at an a of 0.05.

After adjusting for potential confounders, there was no difference in mortality if initially managed as a code tauma activation (OR 1.57, CI, 0.53–4.27, *P* = .42). Independent predictors of mortality included age (OR 1.03, CI, 1.01–1.05, *P* = .01), NIHSS (OR 1.10, CI, 1.04–1.15, *P* < .001), early imaging < 20 minutes (OR 0.44, CI, 0.22–0.90, *P* = .02), and hemorrhagic stroke type (OR 2.87, CI 1.42–5.80, *P* = .003) (Table 4).

## DISCUSSION

Our study highlights that patients with acute stroke who are initially evaluated under trauma activation protocols experience significantly longer times to brain imaging and worse outcomes compared to those triaged through dedicated stroke pathways. While patients arriving as CTA had minimal injuries, they had nearly double the time to imaging compared to those arriving as CSA. Multivariate analysis confirmed that delayed imaging and hemorrhagic stroke were independently associated with mortality, rather than the trauma activation pathway itself. This suggests that operational delays rather than injury burden may be contributing to poor outcomes; however, causality cannot be inferred from this retrospective study.

Our most notable finding was the difference in time to brain imaging, with a mean of 47.5 minutes in the CTA group vs 24.8 minutes in the CSA group. While there are limited studies comparing stroke outcomes based on team activation, Madhok et al found no difference in time to CT for patients seen as a trauma activation compared to those evaluated in other pathways.^13^ However, one institution recognized the potential for delay in management of patients evaluated in a trauma pathway and created a novel pathway called “STRAUMA,” which evaluates patients as a dual code trauma and code stroke and put a heavy emphasis on early neurology evaluation and brain imaging. Lee et al evaluated 580 patients at a Level I trauma center, 469 stroke alerts and 111 STRAUMA alerts. They still found increased time to brain imaging even with their novel STRAUMA alert pathway (23.1 minutes in the STRAUMA group vs 16.9 minutes in the stroke group, *P* < .001). Similar to our results, they also found decreased use of thrombolytics (13.5% in STRAUMA vs 27.9% in stroke group, *P* <. .001) and higher mortality in the STRAUMA group compared to the code stroke group (14.4% vs 6.0%, *P* = .003).^14^

Importantly, Lee et al compared STRAUMA only with code stroke alerts. To assess operational value, future evaluations should also benchmark STRAUMA against standard CTAs as the dual alert could still provide benefit for patients with atypical symptoms brought in as a traditional trauma alert. Just as ED crowding has been shown to adversely affect door-to-imaging times and, therefore, overall outcomes for stroke patients, it is likely that aspects of the trauma evaluation at many institutions contribute to slowing of critical imaging in stroke patients.^15^ While some patients may require initial stabilization and airway management, it is unlikely this played a large role in our patient population given the minimal traumatic injuries noted in the CTA group.

In hemodynamically stable patients with minimal external injury yet focal neurologic deficits, several routine steps in the trauma bay can add clinically significant time to CT, such as positioning for chest and pelvis radiographs, possibly adjusting the plate for adequate radiographs, extremity plain films, and bedside ultrasound. These can affect transport time to the CT scanner and even change the patient’s priority for the CT scanner if higher acuity patients arrive in that time interval. Many of these tasks are deferrable until after head CT without compromising trauma evaluation as long as the primary survey is complete and the patient remains hemodynamically stable. Operational pathways that prioritize direct to CT after primary survey, with parallel neurology notification, may shorten door-to-imaging time while preserving patient safety.

It seems that in nearly half of CTA patients, the trauma team recognized the possibility of an underlying neurologic event, as 42% underwent both typical non-contrast trauma CT and CT angiography of the head and neck during their initial imaging session. This dual-imaging approach suggests that the team had early suspicion for stroke, even while proceeding through a trauma activation pathway. Incorporating CT angiography in such cases may help identify large vessel occlusions and expedite reperfusion therapies. Future protocols could consider establishing a low threshold for adding CT angiography of the head and neck in trauma patients with unexplained neurologic deficits or minimal external injuries, as this strategy may reduce delays in diagnosis without significantly altering trauma workflows.

When intervention was performed, we found no statistically significant difference in time to interventions in the CTA compared to the CSA group. However, we did find a large disparity in the use of thrombolytics in patients with ischemic stroke: only 3% of CTA patients with ischemic stroke received thrombolytics compared to 25% in the CSA group. Despite the proven benefits of thrombolytic therapy in acute ischemic stroke both in acute and long-term outcomes, patients arriving as a CTA are receiving this treatment less frequently.^16^ Documented contraindications to thrombolytics, such as unknown last known well time, factor Xa use, or NIHSS < 6 accounted for 22.4% of ischemic stroke patients in the CTA group who did not receive thrombolytics. However, this does not fully explain the treatment gap compared to CSA patients, suggesting that delays in imaging or reluctance to administer thrombolytics in the setting of trauma may also contribute to missed therapeutic windows. These findings underscore two modifiable targets for trauma-activated stroke patients: expediting imaging, and actively identifying last known well time early in the trauma evaluation. Both may improve access to time-sensitive reperfusion therapies.

Our CTA patients had worse outcomes overall, including higher mortality and less frequent discharge home compared to skilled nursing facility or hospice. However, on multivariate analysis, evaluation as a code trauma was not independently associated with mortality. Age, NIHSS score, stroke type, and early imaging were independently associated with mortality. These have been well-studied in the literature, with studies confirming worse outcomes in older age, hemorrhagic stroke, higher NIHSS score, and delayed imaging.^17^–^20^ Our CTA cohort contained a higher proportion of hemorrhagic stroke. We hypothesize this reflects non-focal presentations typical of hemorrhagic stroke (such as syncope, severe headache, or vomiting) which, especially in the presence of minor external injuries, may more likely prompt a trauma activation than the classic unilateral deficits of ischemic stroke.

While we cannot intervene on the type of stroke a patient has, their age, or the severity of the NIHSS score, we did observe system-level opportunities for improvement. For patients with minor external injury yet focal neurologic deficits, establishing a stroke-in-trauma order set (including CTA head/neck along with non-contrast CT head), early capture of last known well time, a parallel notification of neurology (such as in the STRAUMA study), or even a direct to CT after primary survey could reduce door-to-imaging times and potentially improve access to reperfusion therapy. Even modest reductions in treatment delays can expect to lower long-term disability, thereby reducing disability-adjusted life years (DALYs).^21^ Future prospective work should track patients’ functional outcomes to enable DALY estimation. Additionally the resource implications of a dual-activation system should be evaluated against other costs such as CT use, ED length of stay, re-imaging rates and intensive care unit and hospital length of stay.

Lastly, prehospital stroke screening likely plays a major role in how patients are triaged and ultimately evaluated. Emergency medical services (EMS) impression can direct otherwise similar patients toward trauma vs stroke pathways, especially when the patient is found down or has minor signs of trauma such as facial abrasions or lacerations. Tools such as the Cincinnati Prehospital Stroke Scale have been validated and shown to be useful in the prehospital setting^22^; however, use and documentation of these tools and EMS prenotification were not captured in our dataset. Regional protocols may also favor trauma activation in borderline cases. These unmeasured prehospital factors could contribute to pathway selection and the observed time metrics. Future work should prospectively capture EMS screening results, pre-notification, and destination criteria, and evaluate whether standardized screening plus criteria-based dual activations can shorten imaging delays while preserving patient safety.

## LIMITATIONS

Our study has several limitations. Our single-center design places limits on the generalizability of the findings. Further, the retrospective design introduced potential selection bias and limits causal inference. As with all registry-based reviews, there was the possibility of coding inaccuracies, which may have influenced the results. Additionally our registry did not include prehospital variables, such as the accuracy of field stroke- screening tools, EMS clinical impressions or prehospital decision-making, all of which could contribute to whether patients were brought in as a code trauma vs code stroke. Additionally, operational factors such as trauma team composition or strict adherence to our trauma algorithms may influence time to brain imaging, but these data were unavailable.

The CSA patients were restricted to 2024 due to data availability, whereas CTA patients spanned 2018–2024; although there were no major institutional changes to stroke pathways during that time, this difference may have introduced temporal bias. Last known well time is not well-documented in many of the CTA patients, which likely represents a confounder. Although we adjusted for several confounders in our multivariate analysis, unmeasured variables such as prehospital decision-making and EMS triage protocols could have influenced activation type. Lastly, we did not assess time to final radiology interpretation, which may have further clarified intervention delays.

## CONCLUSION

In patients with acute stroke, initial triage as a trauma activation was associated with significant delays in brain imaging and markedly lower rates of thrombolytic administration, despite a low burden of traumatic injury. Although trauma activation alone was not independently associated with mortality, delays in imaging and a higher incidence of hemorrhagic stroke in this group were strongly associated with mortality. These findings highlight an important association between activation pathway and time-sensitive care, while acknowledging that stroke and trauma activations represent inherently different patient populations. Future work should focus on defining safe, evidence-based strategies to streamline imaging for suspected neurologic emergencies in trauma patients, while preserving the essential elements of trauma evaluation. Additional multicenter studies are needed to determine whether modifications to activation algorithms or dual-alert models can reduce delays without introducing unintended harm.

## Figures and Tables

**Table 1:** Characteristics of patients treated for cerebrovascular accident after code stroke activation vs code trauma activation.

Characteristic	Code stroke (n = 208)	Code trauma (n = 198)	*P*-value
Age, years, mean	70.3	75.3	<.001
Male %	59.6%	52.5%	.63
Stroke type			<.001
Ischemic	178 (85.6%)	111 (56.1%)	
Hemorrhagic	30 (14.4%)	87 (43.9%)	
Mode of Arrival			<.001
Ambulance	165 (79.3%)	185 (93.4%)	
Private Vehicle	36 (17.3%)	8 (4.0%)	
Transfer	7 (3.4%)	5 (2.5%)	
NIHSS, mean	9.67	14.44	<.001

*NIHSS*, National Institutes of Health Stroke Scale.

**Table 2:** Injury pattern of patients treated for cerebrovascular accident after code trauma activation.

Characteristic	Code trauma(N = 198)
Mechanism, n (%)	
Fall	174 (87.9%)
Found down	18 (9.1%)
MVC	6 (3.0%)
Activation level	
Level 1	81 (40.9%)
Level 2	83 (41.9%)
Level 3	34 (17.2%)
Injuries	
Extremity fractures	5 (2.5%)
Spinal fractures	5 (2.5%)
Pelvic fractures	0
Splenic laceration	2 (1.0%)
Facial abrasions/hematomas	66 (33.3%)
ISS, mean (IQR)	3.3 (3.0)
GCS total, mean	11.7
Initial dual imaging*	84 (42.4%)

* Patients underwent non-contrast trauma scans and stroke-protocol scans during initial imaging session

*GCS*, Glasgow Coma Scale*; ISS*, Injury Severity Score; *IQR*, interquartile range; *MVC*, motor vehicle collision.

**Table 3:** Outcomes of patients treated for cerebrovascular accident after code stroke activation vs code trauma activation.

Outcome	Code stroke (n = 208)	Code trauma (n = 198)	*P*-value
Time to brain Imaging, mean, minutes	24.8	47.4	< .001
Thrombolytics, n (%*)	46 (25.8%)	4 (3.6%)	< .001
Door-to-thrombolytics time, mean in minutes	43.7	50.3	.19
Thrombectomies, n (%*)	35 (19.7%)	22 (19.8%)	.13
Door-to-puncture time, mean	82	98	.18
LOS, mean, days	9.0	9.55	.29
Discharge disposition, n (%)			< .001
Home	89 (42.8%)	46 (23.2%)	
SNF	73 (35.1%)	78 (39.4%)	
Hospice	3 (1.4%)	13 (6.6%)	
AMA	11 (5.3%)	4 (2.0%)	
Mortality, n (%)	25 (12.0%)	48 (24.2%)	<.001

* Percentages are for patients with ischemic stroke in each category (178 for code stroke activation, and 111 for code trauma activation).

*AMA*, against medical advice; *LOS*, length of stay; *SNF*, skilled nursing facility.

**Table 4:** Multivariable logistic regression analysis for risk of mortality for patients with acute stroke.

Risk factor	OR	CI	P-value
Age (Years)	1.03	1.01–1.05	.01
<2 Comorbidities	0.88	0.33–2.36	.81
NIHSS	1.10	1.04–1.15	< .001
Early imaging (<20 minutes)	0.44	0.22–0.90	.02
Hemorrhagic stroke (vs ischemic)	2.87	1.42–5.80	.003
Arrival as Code Trauma	1.57	0.53–4.72	.42

*NIHSS*, National Institutes of Health Stroke Scale; *OR*, odds ratio.
